# The Flipping Suture Technique for Reduction of Chronic Incarcerated Bucket-Handle Meniscus Tears

**DOI:** 10.1016/j.eats.2024.103327

**Published:** 2024-12-03

**Authors:** Francesco Bosco, Alessandro Ghirri, Domenico Lewis Battaglia, Fortunato Giustra, Alessandro Massè, Ajay Thakur

**Affiliations:** a Department of Precision Medicine in Medical, Surgical and Critical Care, University of Palermo, Palermo, Italy; b Department of Orthopaedics and Traumatology, G.F. Ingrassia Hospital, Palermo, Italy; c Department of Orthopaedics and Traumatology, Ospedale San Giovanni Bosco—ASL Città di Torino, Turin, Italy; d Department of Orthopaedics and Traumatology, University of Turin, Turin, Italy; e Consultant Sports & Arthroscopy Surgeon, Star Hospitals, Hyderabad, Telengana, India

## Abstract

Meniscal tears are the most common and most treated injuries of the knee. In the recent past, meniscectomy was considered the treatment of choice for meniscal lesions. However, this practice is progressively being discontinued in favor of meniscal repair, which has shown high success rates. Several techniques are currently used individually or in association to treat bucket-handle meniscal tears. The inside-out technique has been considered the gold standard for this kind of lesion, especially if the posterior horn of the meniscus is involved. This article aims to describe a reproducible surgical technique for chronically incarcerated bucket handle meniscal tears that consist of an outside-in reduction suture for meniscal eversion, the “flipping suture”, followed by additional standard meniscal repair sutures.

Meniscal tears are the most common and most treated injuries of the knee. The treatment of these lesions depends on the tear’s localization, which may be identified through Cooper’s classification (Fig 1).^1^ Recently, meniscectomy was considered the treatment of choice for meniscal lesions. However, this practice is progressively discontinued in favor of meniscal repair, which has shown success rates between 85% and 90%.^2^ Bucket-handle meniscal tears (BHMT) can be considered a displacement of longitudinal tears, which causes locking and pain in the knee due to migration in the intercondylar notch.^3^ Meniscal repair surgery consists of three different techniques: all-inside, inside-out, and outside-in. The first is the most adopted worldwide for its effectiveness and efficiency, aided by technological advancement supporting this procedure. The second and third are currently used for displaced BHMT or body and posterior horn tears, the latter being preferred for anterior horn lesions.^4^ BHMT has a higher overall failure rate, as opposed to simple tears. This trend increases in chronic incarcerated tears, for which the failure rate is estimated at 47%, rising to 80% in complex tears.^5^ In recent years, biological augmentation, in the setting of meniscal repair, has gained success among surgeons; bone marrow stimulation and microperforations, platelet-rich plasma, and stem cell therapy are now being extensively adopted to ensure a better healing process of the menisci and joint cartilage. Several surgical techniques have been proposed to help surgeons accomplish an effective meniscal repair.

**Fig 1:**
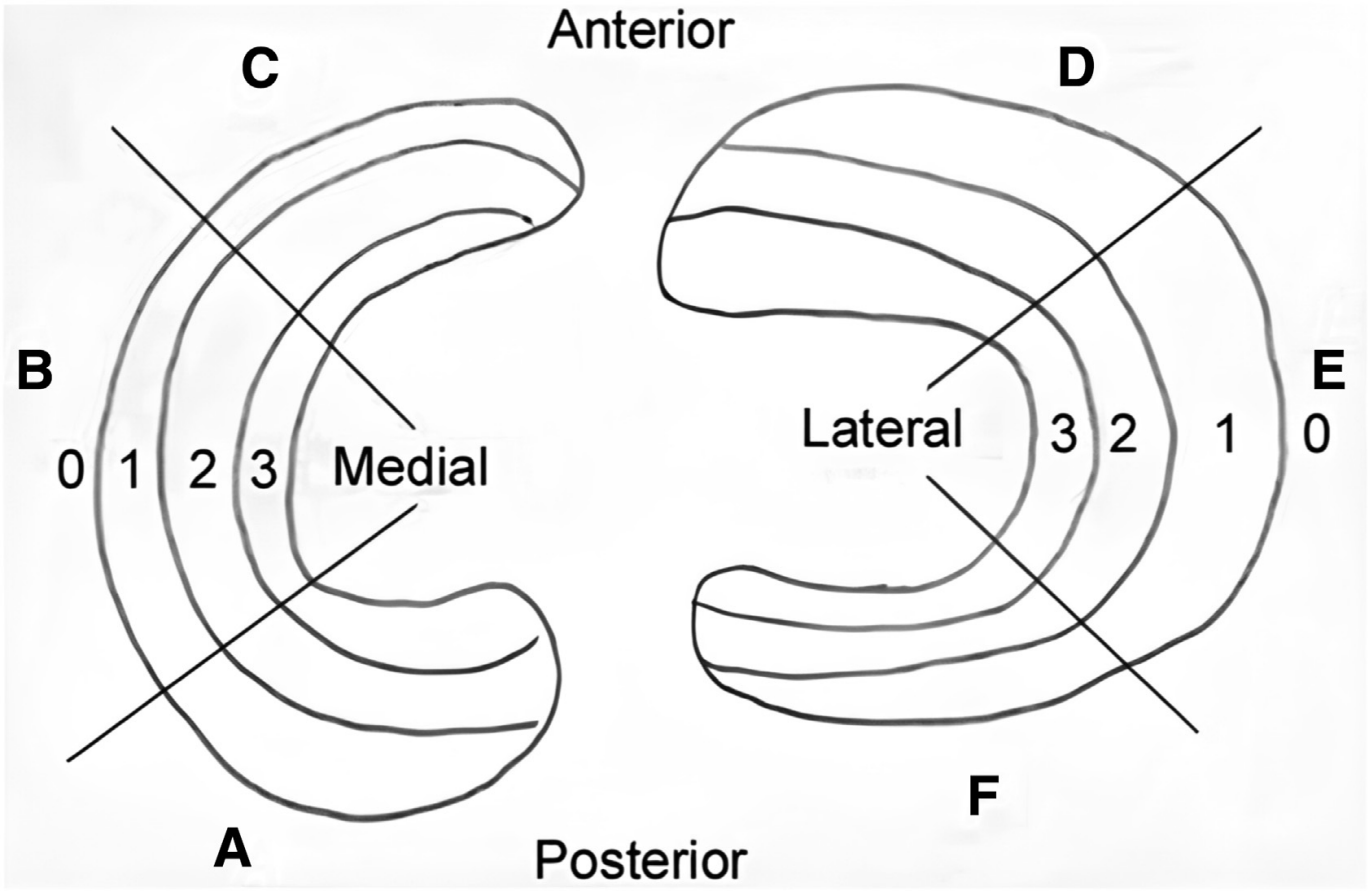
Cooper's Classification of meniscal tears: The medial meniscus is identified with letters (A) Posterior horn of the medial meniscus. (B) Body of the medial meniscus. (C) Anterior horn of the medial meniscus. The lateral meniscus is identified with letters D–F. (D) Anterior horn of the lateral meniscus. (E) Body of the lateral meniscus. (F) Posterior horn of the lateral meniscus. The four circumferential zones are 0 for the meniscal-capsular junction, 1 for the outer third, 2 for the middle third, and 3 for the inner third.

This article aims to describe a reproducible surgical technique for chronically incarcerated bucket handle meniscal tears. It consists of an outside-in reduction suture for meniscal eversion, followed by standard meniscal repair techniques.

## Surgical Technique

The video illustrates the surgical technique in detail (Video 1).

### Preoperative Assessment

The patient was evaluated preoperatively with clinical examination and standard knee x-rays. The patient experienced knee instability, stiffness, swelling, and pain in the affected compartment, locking, and inability to achieve full extension of the knee. Long leg radiographs should be considered if, upon clinical examination, a lower limb deformity is suspected. Magnetic resonance imaging (MRI) is mandatory for a complete preoperative assessment to assess the morphology of the tear and its complexity, possible associated lesions, such as cruciate ligament tears, ramp, or meniscal root lesions and chondral injuries.

### Patient’s Positioning and Anesthesia

The procedure is conducted under spinal or general anesthesia. The patient is placed in a supine decubitus position with a pneumatic tourniquet applied around the proximal thigh of the affected limb. Side support is positioned at the distal third of the thigh as a pivot point for valgus stress maneuvers during the surgery. A side pad is also placed near the contralateral greater trochanter to enhance stability and reduce pelvic tilting during valgus strain. The leg is then left hanging over the edge of the operating table during the arthroscopic procedure. The affected limb may be positioned on a leg holder, removing the distal portion of the operating table if no associated procedures are needed on the patient.

### Diagnostic Arthroscopy

A standard arthroscopic examination is performed through anterolateral (AL) and anteromedial (AM) portals. The patellofemoral joint is evaluated for abnormal patellar tracking or chondral damage. The central pivot is then thoroughly examined and palpated to ascertain its integrity and tension, as cruciate ligament tears are often associated with meniscal tears. The medial and lateral compartments are fully scouted for meniscal and root lesions and to assess cartilage joint degeneration (ICRS scale). Ramp lesions are examined through an anterior view from the medial compartment and a trans-notch view. An accessory posteromedial portal may be considered to assess possible ramp lesions. In case of a medial meniscus tear with low visibility of the medial compartment, the surgeon should feel a release of the medial collateral ligament through a pie-crust technique.^6^

### Surgical Note—Step by Step

Once the bucket-handle fragment is identified, the surgeon carefully attempts its reduction using a probe or blunt trocar. If successful, the tear site is debrided with 4.5-mm shaver blades, followed by needling at the meniscal-capsular junction. The surgeon may then proceed with the meniscal repair technique of choice. Scar tissue around the displaced tear fragment may prevent reduction. Therefore, accurate rasping and debridement need to be performed before the reduction maneuver. This meticulous approach ensures the best possible outcome for the patient.

**Fig 2:**
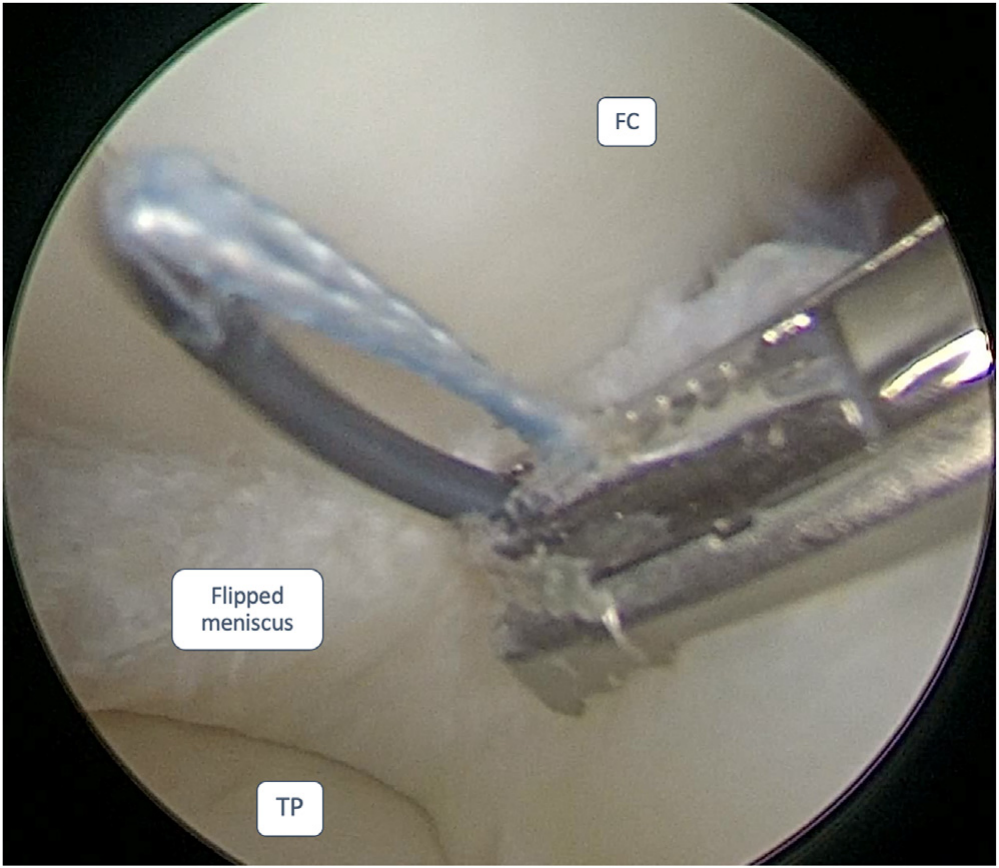
Anterolateral portal view of a medial meniscus bucket-handle tear of a left knee. The surgeon is positioning a suture wire through an antegrade self-retrieving suture device, applying a suture at the base of the meniscus fragment. The patient was placed in a supine decubitus position. The leg was then left hanging over the edge of the operating table during the arthroscopic procedure. FC, femoral condyle; TP, tibial plateau.

If the reduction is not successful, hence the necessity of meniscal-reduction suture techniques, the authors advise using an antegrade self-retrieving suture device (Scorpion Suture Passers; Arthrex, Naples, FL) and applying a suture at the base of the meniscus fragment (Fig 2). The aforementioned suture needs to be positioned at the base of the fragment, along the femoral condyle, where the bucket-handle tear is flipped in the intercondylar notch.

**Fig 3:**
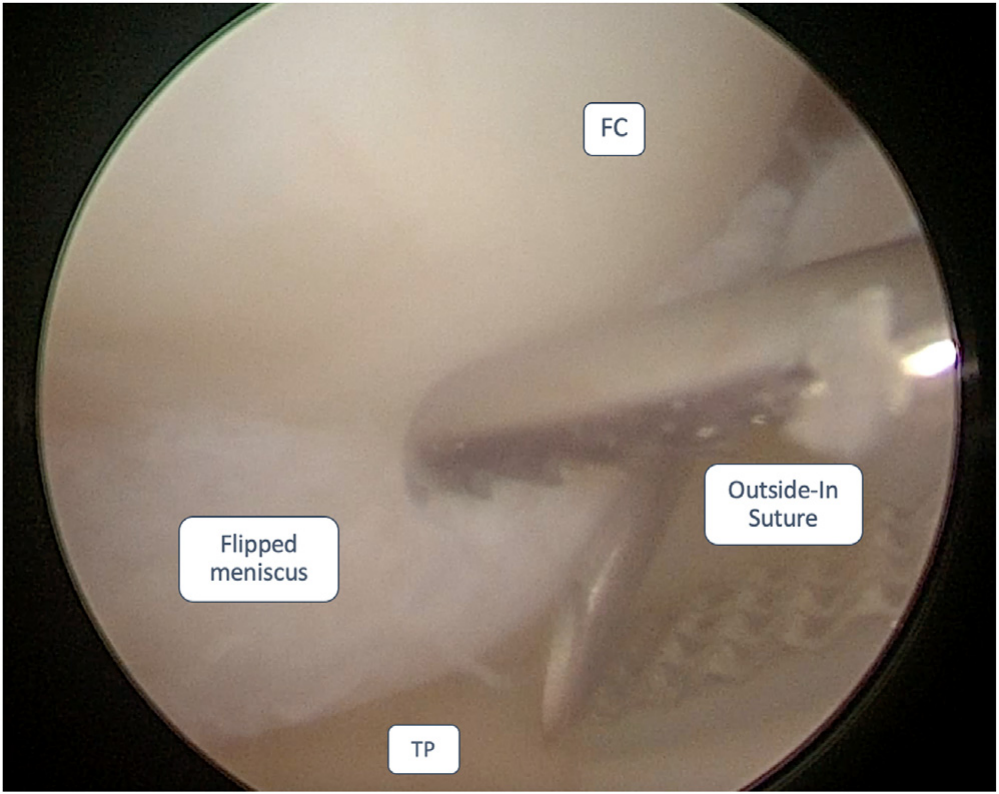
Anteromedial portal view of a medial meniscus bucket-handle tear of a left knee. Outside-in technique suture shuttle positioning at the meniscal-capsular junction. The patient was placed in a supine decubitus position. The leg was then left hanging over the edge of the operating table during the arthroscopic procedure. FC, femoral condyle; TP, tibial plateau.

An 18-gauge spinal needle is inserted at the meniscal-capsular junction, where the meniscal body is normally seated, to insert a nylon suture shuttle, which is then retrieved through the AM portal (Fig 3).

**Fig 4:**
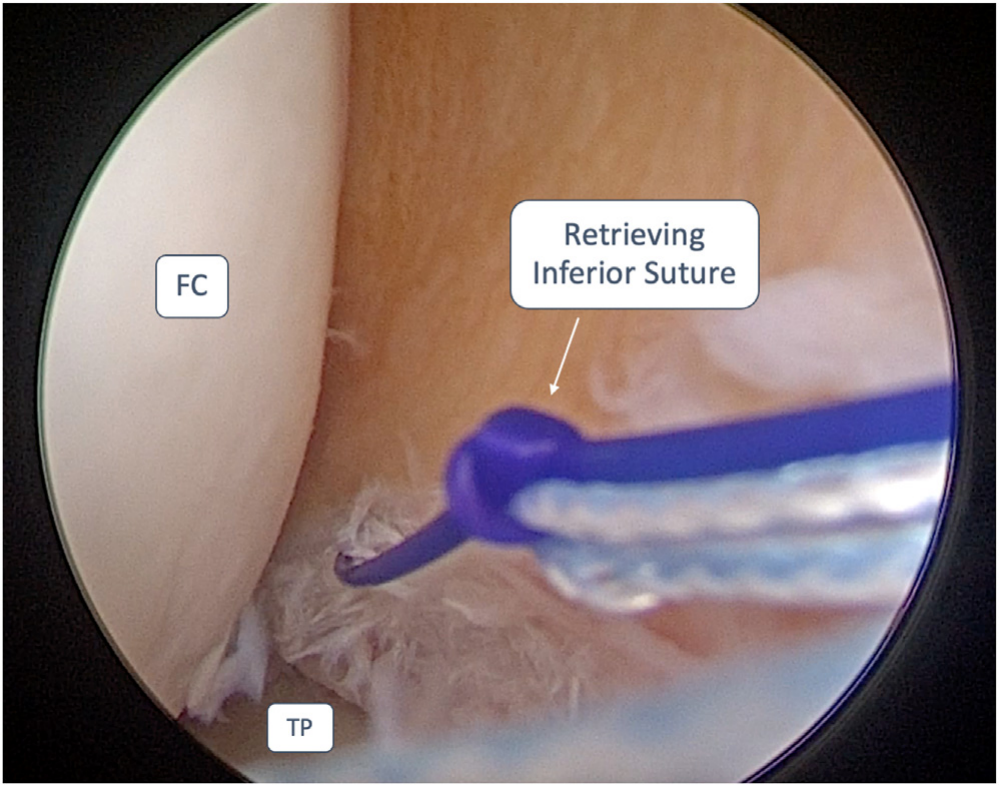
Anterolateral portal view of a medial meniscus bucket-handle tear of a left knee. To obtain a “Flipping Stitch”, the inferior end of the suture wire is retrieved at the meniscal-capsular junction with an outside-in technique. This step is repeated for the superior end of the suture wire. The patient was placed in a supine decubitus position. The leg was then left hanging over the edge of the operating table during the arthroscopic procedure. FC, femoral condyle; TP, tibial plateau.

**Fig 5:**
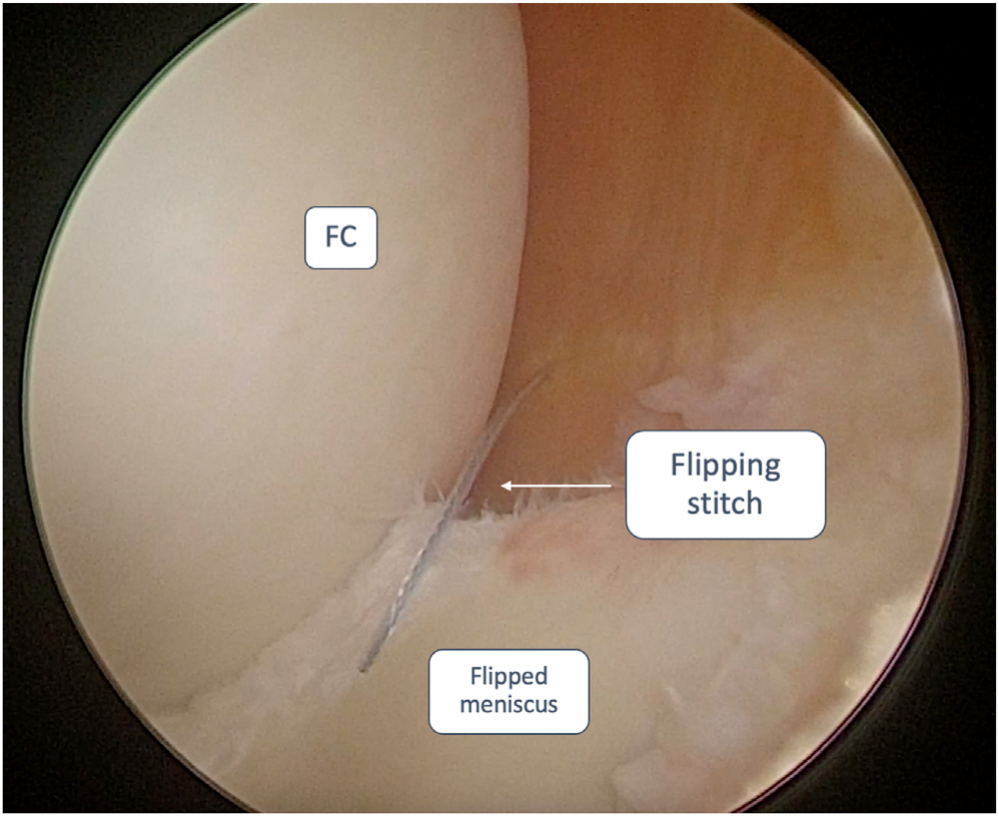
Anterolateral portal view of a medial meniscus bucket-handle tear of a left knee. Both ends of the suture wire are retrieved out of the skin, and while maintaining constant tension on the threads, the everted meniscal bucket-handle fragment is flipped. The patient was placed in a supine decubitus position. The leg was then left hanging over the edge of the operating table during the arthroscopic procedure. FC, femoral condyle.

The “flipping-stitch” is achieved using an outside-in technique, retrieving the inferior suture end first (Fig 4), followed by the superior suture, using the suture shuttle previously inserted. Once both the suture ends are retrieved from the skin, the everted meniscal bucket-handle fragment is flipped, maintaining a constant tension on the threads and a gentle varus or valgus stress with a blunt trocar or probe (Fig 5). Once complete reduction of the fragment is achieved, maintain minimum tension during stressing maneuvers to avoid redisplacement of the fragment. The fragment is stabilized using all-inside or inside-out suturing techniques (FiberStitch or ZoneNavigator system with 0.9-mm SutureTape, Arthrex, respectively). Sutures can be configured in either vertical or horizontal mattress patterns to ensure adequate repair stability and anatomical fixation. When employing the outside-in technique, a vertical suture should be positioned at the terminal end of the tear on the anterior horn of the meniscus, followed by a second vertical suture placed on the posterior horn. These sutures are strategically placed to prevent the anterior and posterior propagation of the meniscal fragment (Table 1).

**Table 1:** Highlights of the Surgical Technique

• Perform diagnostic arthroscopy to rule out concomitant intra-articular injuries and assess the extent of the meniscal tear.
• Visualize and attempt to reduce the bucket-handle fragment using gentle manipulation techniques.
• If the reduction is not achieved, employ an antegrade self-retrieving suture device to stabilize the fragment. Apply a suture at the base of the meniscus fragment, where it turns along the femur condyle.
• Use an 18-gauge spinal needle to retrieve the inferior suture first, followed by the superior suture using the outside-in technique.
• Once both suture ends are retrieved, maintain constant pull on the threads while pushing the meniscus fragment from the inside using a probe or trocar.
• Ensure successful reduction of the fragment without causing damage to the femur condyle or the meniscus fragment.
• With successful reduction achieved, repair the remainder of the meniscus using appropriate techniques.

### Postoperative Protocol

After surgery, the patient is allowed 0–90° range of motion for 4 weeks. Full extension needs to be achieved in the first 15 postoperative days. The surgeon should consider 2 weeks of immobilization in a knee brace locked in full extension for patients with a preoperative locked knee and an extension deficit. Isokinetic exercises should be started immediately from postoperative day 1. Non–weight-bearing is advised for 4 weeks postoperatively, followed by protected weight-bearing for the following 2 weeks. A broader range of motion (0–120°) is allowed from the fourth postoperative week. Range of motion >120°, kneeling, and squatting should be avoided up to the fourth postoperative month. The patient is followed up at 2 weeks, 1 month, 6 months, and 1 year. Return to sports activities is allowed, depending on the physiotherapy achievements of the patient.

## Discussion

Bucket-handle tears are complex meniscal injuries, constituting about 10% of cases.^7^ MRI is essential for diagnosis, revealing signs like the double posterior cruciate ligament (PCL) and flipped meniscus sign. Missing meniscal bodies in sagittal cuts indicate the absence of the bow tie sign.^7^^,^^8^

Long-term studies show increased arthritis after partial meniscectomy.^8^ Logan et al.^9^ found higher peak contact pressures after medial BHMT resection during ACL reconstruction. Patients who undergo BHMT repair have less knee pain and better outcomes than those with meniscectomy.^8^, ^9^, ^10^

The literature describes preferred treatment techniques, including inside-out and all-inside repair techniques.^2^^,^^11^^,^^12^ Studies show both techniques effectively restore meniscus biomechanics, with no significant difference in contact area or pressure.^12^^,^^13^

Regarding clinical outcomes, good long-term results have been demonstrated for BHMT repairs, both inside-out and all-inside techniques. Samuelsen et al.^14^ showed clinical success rates of 80% for inside-out and all-inside repairs. They found no difference in clinical success rates, clinical outcome scores, or complication rates between the two techniques. Cetinkaya et al. reported a clinical healing rate of 77% and a radiological healing rate of 81% with MRI for 26 patients, with chronic BHMT repaired using all-inside or inside-out techniques at 6 months postoperatively.^15^

BHMT is often displaced and unstable, requiring inside-out suture repair with or without all-inside repair.^7^^,^^12^^,^^16^, ^17^, ^18^, ^19^ The rationale for inside-out suture repair is that, unlike all-inside meniscal repair, it permits accurate reduction, stabilization, and coaptation of the tear edges.^16^ The disadvantages of using an inside-out suture repair include the need for skin incisions either posterolaterally or posteromedially, where there is a risk of common peroneal nerve and saphenous nerve palsy, respectively. Overall, the risk of infections or neurovascular complications has been reported as high as 21%^12^ (Table 2).

**Table 2:** Pearls and Pitfalls

Pearls
• Ensure the patient is well positioned with lateral support and a side pad near the contralateral greater trochanter to facilitate stress maneuvers and ensure proper visualization of the menisci.
• In case of a medial meniscal bucket-handle tear, perform a release of the medial collateral ligament using the pie-crusting technique. If a ramp lesion is suspected, perform the posteromedial portal.
• Scar tissue around the displaced tear fragment may prevent reduction; therefore, accurate rasping and debridement must be performed before the reduction maneuver.
• If the initial reduction attempt is unsuccessful, prompting the need for meniscal-reduction suture techniques, the authors recommend utilizing an antegrade self-retrieving suture device to apply a suture at the base of the meniscus fragment
• After an outside-in suture reduces the meniscal fragment, apply minimal traction during stress maneuvers to prevent its redisplacement.
Pitfalls
• Errors in patient positioning and the omission of placing the side pad near the contralateral greater trochanter could lead to difficulties performing arthroscopy and visualizing intra-articular lesions.
• In the presence of a medial meniscus lesion, refraining from performing a release of the medial collateral ligament could hinder the accurate diagnosis of the injury.
• If two vertical stitches are not placed at the end of the tear on the meniscus’s anterior and posterior horns using an outside-in technique, there is a risk of lesion propagation.
• Granting early weight-bearing on the operated lower limb could lead to failure of the meniscal suture.

In conclusion, the technique described in this study involves using an antegrade self-retrieving suture device along with an outside-in stitch. The goal is to enhance control over reducing the meniscal lesion, which may not be entirely achievable with traditional all-inside stitches alone. Additionally, the method aims to fixate the lesion using all-inside sutures, thereby reducing risks and complications associated with the inside-out technique.

## Disclosures

The authors declare (F.B., A.G., D.L.B., F.G., A.M., A.T.) that they have no known competing financial interests or personal relationships that could have appeared to influence the work reported in this article.

## Supplementary Data

Video 1 In this video, we will describe a surgical technique for the treatment of chronic bucket handle meniscal tears. This technique consists of using an outside-in “flipping suture” followed by a standard meniscal stabilization repair. The patient in this video is a 17-year-old male who is active in sports like football and cricket, who suffered by a dislocation trauma in the left knee. • Complained of instability and a locked knee in 20° flexion. • Grade 3 anterior drawer test • Grade 1 Pivot and Lachman’s test • ROM 20° to 100° • McMurray test could not be elicited. • An MRI scan confirmed a complete ACL tear and a Bucket-handle medial meniscus tear. • Perform diagnostic arthroscopy through standard anterolateral and anteromedial portals. undergoing graduation • Visualize and attempt to reduce the bucket-handle fragment with a blunt trocar. This technique is advised in the case of unsuccessful reduction of the bucket-handle tear. Using an antegrade self-retrieving suture device, the surgeon applies a suture at the base of the meniscus fragment. The surgeon inserts an 18-gauge spinal needle at the meniscal-capsular junction, where the meniscal body is normally seated, in order to insert a nylon suture shuttle that is then retrieved through the anteromedial portal. The “flipping-stitch” is achieved using an outside in technique, retrieving the inferior suture end first. This step is repeated for the superior suture. Attempt reduction again with a blunt trocar or a probe, gently maintaining pressure on the outside in a flipping stitch. In this way, the surgeon may maintain the reduction of the meniscal fragment and continue surgery with additional all-inside stabilizing sutures. It is recommended to proceed with meniscal repair surgery with a combination of horizontal and vertical sutures.
